# Tentacular Faces: Race and the Return of the Phenotype in Forensic Identification

**DOI:** 10.1111/aman.13385

**Published:** 2020-05-06

**Authors:** Amade M'charek

**Affiliations:** ^1^ Department of Anthropology University of Amsterdam Netherlands

## Abstract

The face, just like DNA, is taken to represent a unique individual. This article proposes to move beyond this representational model and to attend to the work that a face can *do*. I introduce the concept of *tentacularity* to capture the multiple works accomplished by the face. Drawing on the example of DNA phenotyping, which is used to produce a composite face of an unknown suspect, I first show that this novel technology does not so much produce the face of an *individual suspect* but that of a *suspect population*. Second, I demonstrate how the face draws the interest of diverse publics, who with their gaze flesh out its content and contours; the face engages and yields an affective response. I argue that the biologization of appearance by way of the face contributes to the racialization of populations. [*race, phenotype, material‐semiotics, facial typologies, forensics genetics, DNA phenotyping*]

## RESUMEN

La cara, justo como el ADN, se toma para representar un individuo único. Este artículo propone ir más allá del modelo representacional y atender al trabajo que una cara puede *hacer*. Introduzco el concepto de *tentacularidad* para capturar los trabajos múltiples logrados por la cara. Basada en el ejemplo de fenotipificar el ADN, el cual es usado para producir una cara compuesta de un sospechoso desconocido, primero muestro que esta tecnología novedosa no produce tanto la cara de un *sospechoso individual*, sino la de una *población sospechosa*. Segundo, demuestro cómo la cara atrae el interés de públicos diversos, quienes, con su mirada, definen su contenido y contornos; la cara envuelve y produce una respuesta afectiva. Argumento que la biologización de la apariencia por medio de la cara contribuye a la racialización de las poblaciones. [*raza, fenotipo, semiótica material, tipologías faciales, genética forense, fenotipificar el ADN*]

“I shouldn't know you again if we *did* meet,” Humpty Dumpty replied in a discontented tone, giving her one of his fingers to shake: “you're so exactly like other people.”

“The face is what one goes by, generally,” Alice remarked in a thoughtful tone.

“That's just what I complain of,” said Humpty Dumpty. “Your face is the same as everybody has—the two eyes, so—” (marking their places in the air with his thumb) “nose in the middle, mouth under. It's always the same. Now if you had the eyes on the same side of the nose, for instance—or the mouth at the top—that would be some help.”

—Lewis Carroll, *The Annotated Alice*

The face—just like DNA—is associated with individuality. It is what makes each of us unique, or so we are told. But what if we would assume the role of Humpty Dumpty? What if we did not take the face for granted and instead opened it up for empirical analysis? For although the face is ubiquitous in everyday life, it has hardly received any attention from cultural anthropologists. This is remarkable given the extensive scholarship on the body (Edmonds 2010; Lock 1993; Mol 2002). It is even more remarkable given the tainted history of anthropology with race science, in which the face has occupied a salient place (e.g., Morris‐Reich 2013, 2016). In this article, I open up the face for analysis to understand how race is done in practice. Doing so might also help us ponder the relation between race and appearance, or the phenotype, another neglected phenomenon.

My aim is not so much to develop a social theory of the face but instead to uncover its specificities in practice—or, following Mol (2002), to become a “praxiographer” of the face.^1^ To shift focus from what the face *represents* and attend to what the face *does*, I offer the metaphor of the tentacle. As sensory organs, tentacles engage with their environment, sensing and feeding on it. Departing from a discussion of the face in the history of physical anthropology, I draw on the case of forensic DNA phenotyping—that is, the inference of visible characteristics of an unknown suspect from DNA to produce a “composite face.” I develop my argument in two steps. First, I show that this technology is not so much aimed at the *individual suspect* but at a *suspect population*: clusters of individuals come into focus for police investigation. When and how does this population become raced, and how does the composite face contribute to this racialization? Second, because this composite face is a generic one, its main value in criminal investigation is its capacity to engage a public that will give it more content and contours while generating clues about the identity of the unknown suspect. For the face to do this work, it needs to attract and affect a public to evoke a response.

My take on face is inspired by the work of Deleuze and Guattari ([1987] 2004). Responding to Levinas, whose ethics relies on the face‐to‐face encounter and in which the face assumes an individual subject, Deleuze and Guattari argue that the face is not so much an individual characteristic, nor does it “assume a prior signifier or subject” (201). The subject is rather an effect (in a Foucauldian sense) of the “abstract machine of faciality” (187). This faciality machine is a material‐semiotic system entwined with notions of normality and deviance in society. As products of this machine‐like process, faces may enact the individual or the collective. Whether it is one or the other is context‐specific and dependent on the distribution of similarities and differences by the abstract machine of faciality. Moreover, the face is not limited to the skull or the head; it spills over to include bodies and even environments. The faciality machine is thus one that affects the entire body, implying that any bodily marker has the capacity to enact the face. Given the importance of faces in Western cultures, so Deleuze and Guattari argue, the social production of face and the facialization of the body provide new ways to understand race and racism. To clarify the process of facialization, Deleuze (1986) distinguishes between the “reflective face” (in which the different parts of the face add up to produce a whole) and the “intensive face” (in which parts of the face become potent, subsuming others and facializing the entire body). I will demonstrate the relevance of this distinction for understanding the work of the face in forensic practice.^2^

In the universe of Deleuze and Guattari, the face is conceptualized as a map and as *surface*, concepts that move our attention from the inner world and the experience of identity to externalities, such as the surface of the body. It is this theoretical gesture (the face as surface) that I want to embrace to think about race in relation to the face in practice. These concerns are of relevance to the discipline of anthropology—and physical anthropology, in particular (see e.g. Sauer 1992, 1993). The discipline, in its interest in human diversity, has historically been entangled with colonialism and racism, contributing to the racialization of difference (Asad 1973; Barkan 1996; Fabian 2010). As Ashley Montagu ([1942] 1945, 27) has famously stated: “‘race’ is, to a large extent, a special creation of the anthropologist.” Nineteenth‐ and early twentieth‐century anthropology indeed represented a turn toward the body as a site for understanding human diversity (e.g., Stocking 1982; Stuurman 2017). Embracing the racial classification and hierarchization of Blumenbach (e.g., Gould [1981] 1994, 1996; Hannaford 1996), the skull became a primary object in attempts to classify humans into racial types. In the virtually obsessive collection and classification practices of anthropologists, not only the skull but also the face, as I show shortly, came to occupy an important role (Morris‐Reich 216; see also Mak, this section). Importantly, this is not a history left behind. These practices not only materialized in books but also in anthropological collections and their classifications, as well as in the measurements, protocols, and tools that populate current‐day laboratories. These sedimented histories continue to play a role in contemporary practices of physical and forensic anthropology, as they do in various technologies of forensic face making, such as facial composite sketching, cranio‐facial reconstruction, and practices of DNA phenotyping. Yet, while history is not simply left behind, it is not merely repeated either (M'charek 2014).^3^

Before introducing these forensic genetic technologies of the face, I will take a step back and discuss more conventional approaches to faces. My example is a well‐known series of faces, produced as *Wandtafeln* by the Swiss physical anthropologist Rudolf Martin (1903a). These prints are in many ways instructive and might help us think through the relation between face, race, and phenotype. While within physical anthropology, and more broadly, the phenotype has been naturalized and mobilized as an instrument for seeing and knowing racial differences, by contrast, I want to suggest that the phenotype has never been solely about the biological, a nature out there. As I show in my discussion of Martin's *Wandtafeln*, the phenotype included both nature and culture. Particularly because what can be observed is highly dependent on situated technologies of vision. I thus argue for a notion of phenotype that goes beyond a strictly biological definition and that takes the practice of seeing seriously. In this way, the phenotype is productively understood as a material‐semiotic object. Attending to this material‐semiotic aspects of face and phenotype, the *Wandtafeln*, and the critical role it played in colonial pedagogies of racial difference, allows me to tease out the operations of the face in DNA phenotyping today: the operations of instructing the viewer about what to take into account, fashioning and typecasting what is viewed, and evoking feelings of interest in the issue at stake.

## MARTIN'S *WANDTAFELN*: FACE AS MATERIAL‐SEMIOTIC OBJECT

In 1903, Rudolf Martin published a selection of twenty‐four faces as single prints, so‐called *Wandtafeln*, for educational purposes with the publisher house Art. Institut Orell Füssli, in Zürich. The prints were accompanied by two other publications: an article in the *Korrespondenzblatt der Anthropologischen Gesellschaft*, the journal of the Anthropological Society, and a book, *Wandtafeln für den Unterricht in Anthropologie, Ethnographie und Geographie*. Martin wrote, “For each panel I have written a short monograph containing the most important literature from which the essentials of the physique and custom of the type in question can be seen” (*Zu jeder Tafel habe ich eine kurze Monographie mit Angabe der wichtigsten Literatur geschrieben, aus der das Wesentliche der Physis und Ergologie des betreffenden Typus ersehen werden mag*; Martin 1903b, 132). The word “ersehen” has become outdated in German today, but it meant both to deduce and to recognize something that presents itself (*etwas Sichbietendes erkennen*). This suggests that the prints were meant to help students arrive at the fact of race by evoking a process of reasoning based on knowledge about difference but also by helping them recognize race immediately—“at face value,” so to speak.

**Figure 1:**
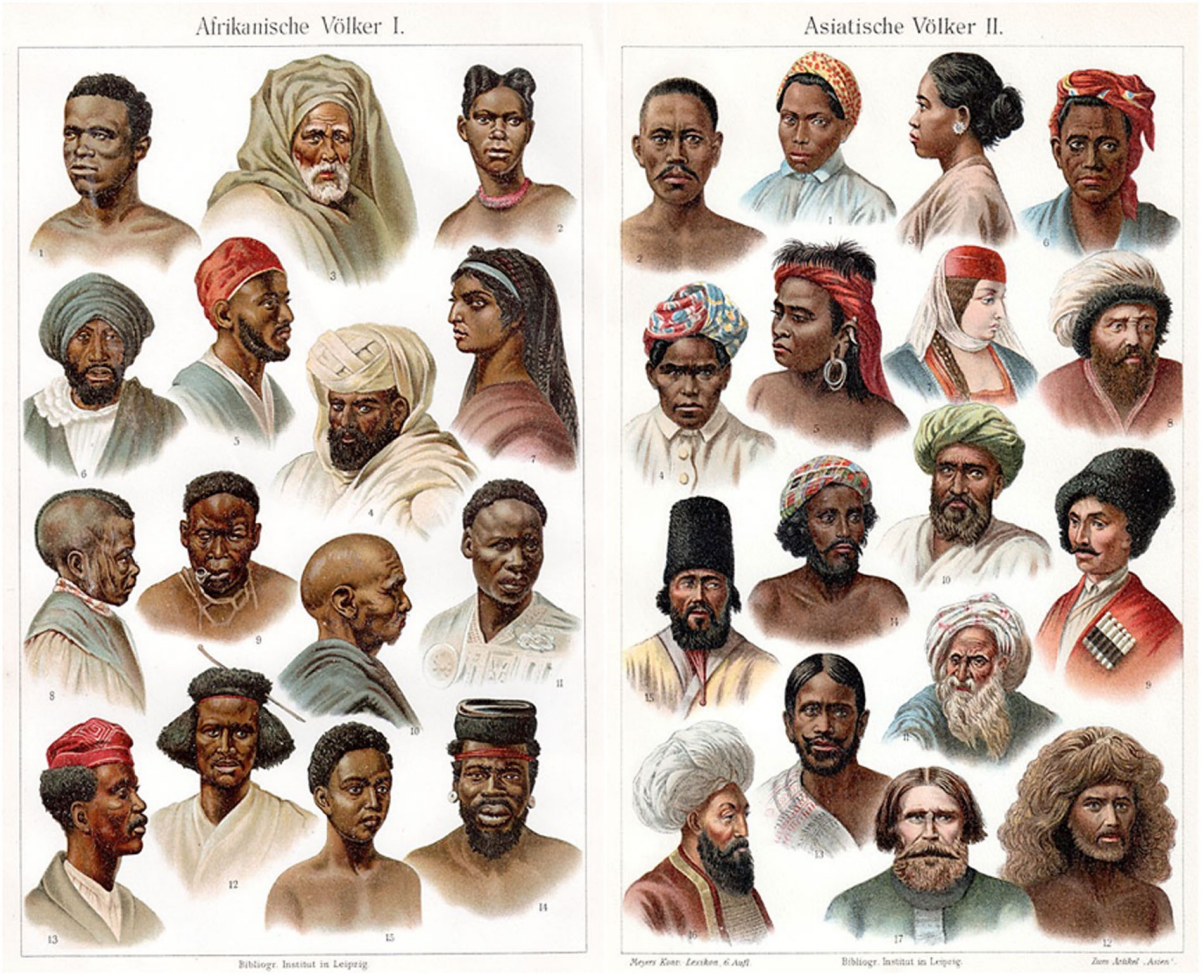
Face value. Meyers Konversations‐Lexikon. (Source: Wikimedia Commons) [This figure appears in color in the online issue]

Both the educational aim of these (and similar) prints as well as the aim to learn to recognize something as it presents itself are instructive here. The prints invite the viewer to look at faces and distinguish between different races (see Figure 1). This is an invitation to learn *types* (*Typus*). But precisely what is the viewer looking at? First, although we may believe that we are merely looking at faces to develop a taste for difference, the prints make clear that the face does not come by itself. It is accompanied by many attributes, such as clothing and hairdos. Facial forms, skin tone, or hair color do not by themselves make racial types. These bodily features are connected to a range of cultural items that together help to produce a racial type.^4^ These prints provide us with an example of race as a material‐semiotic object (M'charek 2013). Race cannot be reduced to the body or parts of it but comes about as a relation between the body (its surface) and various other entities. As Martin writes, the racial type is to be learned from “Physis und Ergologie,” from its physique and its custom.

Second, race comes about not only through a relation between the face and the surrounding attributes but also through the relation between one face and another. Juxtaposing many different faces, proximate and distant from one another, in a more or less similar format produces an illusion of objectivity. It is a version of “trained judgment” (Daston and Galison 2007) that produces a standardized way of *observing* that seems to eliminate the subjective effect of the viewer. The prints instruct and guide the gaze of the viewer yet obscure the work of instruction (the work of *ersehen*). They thus present racial differences as matters of fact.

While the prints obscure their instructive and educating work, attending to that very work helps us to see that the face and bodily appearance—the phenotype—are not simply qualities of the body but are dependent on our technologies of vision (Haraway 1991b). Seeing is always situated and always mediated through knowledge, technologies, devices, theories, ideologies, and so on. The prints, and their capacity to instruct us as viewers, operate as technologies of vision, devices that guide our gaze and help us develop a taste for difference and sameness. The learning aspect of the prints was, as indicated, precisely their purpose, as they were meant to be part of the educational program of the anthropologist. But they were not solely aimed at students of anthropology. They were mass‐produced and widely distributed in Europe and provided a way for the general public to get to know human diversity. The collage of faces above has been reproduced in the major German‐language encyclopedia *Meyers Konversations‐Lexikon*, the sixth edition of which was published between 1902 and 1908. This edition was the most successful, selling around 240,000 sets, a success that was halted by World War I. As part of the encyclopedia and beyond, the prints have brought into being a collective gaze and have thus been a crucial technology of vision.

This example shows that the face does not simply represent an individual, or a racial type, but is active and involved in multiple works. The face *instructs* the viewer on how to see racial differences. But it also *fashions* and *typecasts* these differences. The face does not stand alone but is connected to various cultural markers. Understanding the work of the face, I suggest, requires that we view race as a relational object. Thus, the face, just like race, is a material‐semiotic object. In what follows, I briefly situate the face in relation to race and genetics and the emerging interest in the phenotype. I then move to the work face is doing in DNA phenotyping practices. First, I focus on how composite faces enact the individual and the collective, and then I attend to these faces in criminal investigations. I show that the work of faces consists not only of *instructing* the viewer and *fashioning* difference but also in *evoking* feelings and interest in criminal cases.

## RETURN OF THE PHENOTYPE

In the field of forensic genetics, as I elaborate below, the face and race are moving center stage. The research and the growing interest in the face contribute to what I call “the return of the phenotype”—that is, the biologization of appearances. But how does this compare with the increasing interest in the interiority of the body, in terms of genes, and with the pivotal role of genetics and genomics today? How does this compare with the commonly heard statement that race does not exist because in genetic terms humans are more than 99.9 percent the same?

When the draft of the human genome was presented to the world in June 2000, it was celebrated as a monument of humanity, and one that spoke to our commonality. This message was brought to us not through a scientific paper but by a group of powerful men gathered at the White House: Bill Clinton (president of the United States), Tony Blair (prime minister of the United Kingdom, via a conference call), Francis Collins (director of the National Human Genome Research Institute), and Craig Venter (chief executive of Celera Genomics). We are, Clinton told us, more “than 99.9 percent the same.”^5^ However, the genetic research that was sparked by this important achievement in life science research, the map of the human genome, turned out to be centered not so much on our sameness but rather on that 0.1 percent of difference. The focus on difference as a site to learn about genetic diseases, genetic genealogy, or forensic genetics turned out to be much more promising.

Large databases have been developed since then through the International Haplotype Mapping Project, the 1000 Genomes Project, and, more recently, the All of Us project, among others.^6^ This focus on difference has itself attracted attention and critique, and thus has become equally present in social science research on genetics and genomics. An important and influential observation in this scholarship is that despite the promise of the common genome, race did not become irrelevant in life science research. Although genetics and genomic research claim to be colorblind or “postracial,” various scholars argue that such research is contributing to the “reinscription of race at the molecular level” (Duster 2006, 428; see also Abu El‐Haj 2007; Fullwiley 2007; Skinner 2006).

Although this process of molecularizing difference is highly important and requires ongoing attention in the context of big data and data‐mining endeavors, here I want to suggest that in the life sciences we are increasingly witnessing *the return of the phenotype*—in other words, the biologization of appearance. The growing interest in the biology of appearance is reconfiguring relations between the individual and the population as well as shaping what race is made to be. The return of the phenotype might lead to the suggestion that genes map neatly onto appearance and, the other way around, that appearance can predict a person's genetic composition or even behavior. It is precisely this assumed causal relation between genotype and phenotype that I problematize. Moreover, with the return of the phenotype, I suggest that race is becoming a matter of sur‐face.^7^ Might it then be that Ashley Montagu's observation—that while physical anthropologists were clinging to the race concept, geneticists had moved beyond it (Visweswaran 1998, 74)—is currently being overturned with an increasing interest in the phenotype? Might this interest in the phenotype risk introducing racial typologies through the front door?

## ATTENDING TO THE PHENOTYPE

The face has been neglected in social science research but so has the phenotype and its relation to race. This has largely to do with the important yet dominant social constructivist approach to race (e.g., Hartigan 2009). This approach is based on the idea that racialized differences are intricate constructions, yet they are built on crude and given phenotypical variations. Specific features such as skin color are taken to present themselves immediately to the viewer. These are “given facts,” or the foundation on which the social constructions of race are built.^8^

This take on the phenotype as a biological substratum of the social construction of race bears some resemblance to the assumed difference between sex (a biological fact) and gender (a social construction; Stepan 1986).^9^ Although the introduction of the concept of gender has been incredibly productive for women's and gender studies, the effect of the sex/gender dichotomy has contributed to a reductionist approach to sex (Fausto‐Sterling 2000). While work focusing on the social construction of gender has contributed to the sophistication of gender as a concept, sex—and by extension, the body—has initially received little attention and has been cast as a matter of fact. Sex thus became the foundational yet not‐so‐relevant Other of gender (e.g., Haraway 1991a; Mol [1987] 2015).

The seeming lack of interest in sex differences was not simply a matter of indifference: it reflects the view that the physical and the biological are politically problematic and not immediately helpful for feminist projects. The growing interest in the materiality of the body and the aim of critical scholarship to move beyond critiquing the monolithic stories of the life sciences and to attend to the diverse practices of doing and knowing sex differences have turned sex into an interesting and relevant object of research in gender and feminist science studies (Mak 2012; E. Martin 1991; M'charek 2005; Richardson 2013).

There is an important parallel here with race. One could say that the social constructivist approach to race has equally trivialized and essentialized the phenotype and has helped to reproduce the matter‐of‐fact‐ness of bodily appearance. However, this idea that some phenotypic markers are simply matters of fact, and somehow also the foundations on which the social construction of racial differences is built, is problematic. As Wade (1997, 15) suggests, referring to the colonial racial science of the nineteenth century, the “physical differences that have become cues for racial distinctions are quite particular ones … [ones] that corresponded to the geographical encounters of Europeans in their colonial histories.” This indicates that we cannot take physical markers for granted. Not only does their persistence in science and society have a history, but also our vision is shaped and primed by the resonances of these histories in present‐day societies. This also means that both the constitution of race and the dynamics of racism are historically situated and that their current manifestations are specific to the diverse histories of slavery, colonialism, empire, and migration.

Physical markers are thus an effect of *ersehen*. They are an effect of a historically dense and situated social and scientific practice of “doing difference.” This prompts us to ask: What are the phenotypical markers of difference in this practice here, and what are they made of? To be sure, these are risky questions to raise in the context of race. Attaching race to the body and its appearances might contribute to the naturalization of race and might fuel received ideas that race is surely to be located right there, in the body and its biology. Here, the lessons learned from Martin's prints are helpful. What they help us see is that the phenotype is not something *in* or *on* the body but rather something *of* the body. The phenotypes that helped to make racial types were enacted as a relation between bodily markers, facial markers, and other cultural entities. The phenotype is thus a *material‐semiotic object* par excellence! With this in mind, I now move to the field of forensic genetics to introduce the emerging technology of DNA phenotyping and to analyze the work of the face in the context of criminal investigations.

## FORENSIC DNA: FROM A TOOL OF IDENTIFICATION TO A TOOL OF INVESTIGATION

Forensic DNA made its appearance in various jurisdictions in the late 1980s, and it soon became the champion technology in criminal investigation. It first made its appearance in the United Kingdom in a family reunification case as a technology to prove the relatedness between a young Guinean man and his mother and siblings. Its potential to establish a genetic link between different profiles foreshadowed its use in criminal investigation, in which it could be used to examine a potential link between a biological trace at a crime scene and a suspect (M'charek 2008; see also Skinner 2018). At first, the technology was merely used to include or exclude a suspect by comparing a biological trace connected to a crime with the DNA profile of a suspect. Although its early days were marred by some controversies, forensic DNA would soon become the gold standard in criminal investigations (e.g., Lynch et al. 2008; M'charek 2008). The advancement of genomic and biotechnological research, novel‐marker kits, and larger databases have contributed to a broadened use of the technology. One of the more dramatic changes was that DNA‐profiling technology was not merely used to include or exclude a suspect but to produce clues about unknown suspects, thereby becoming a technology to generate a suspect.

There are various techniques that help to produce hints about the identity of the unknown suspect. One can run a DNA‐database comparison searching for full matches between a crime scene stain and DNA profiles in the database. A DNA dragnet can be commissioned, in which members of the (assumed to be) relevant population are invited to “donate” DNA to rule them out as suspects. A third possibility, “familial searching,” involves near matches with someone in the database or in the sampled population, which indicate that person is a possible relative of the suspect. The person whose DNA is in hand will lead the way to the unknown suspect (Williams and Johnson 2005). A fourth way to produce clues about an unknown suspect is DNA phenotyping, the inference of facial characteristics from DNA.

DNA phenotyping is an emerging field as well as a “promissory science” (Brown 2003; Skinner 2018; see also Fortun 2005). Proponents of the technology promise, as we will see below, no less than a photo of the unknown suspect. In practice, DNA phenotyping currently allows for the inference of the sex of the unknown suspect and for an estimate about their biogeographic ancestry and their eye, hair, and skin color (Kayser 2015).^10^

## THE PROMISE OF THE FACE

There is an ironic coincidence between the presentation of the human genome in June 2000 and DNA phenotyping. At the very moment when the human genome was presented to the world as a testament to human commonality and as the evidence that race did not exist, in the Netherlands the high‐profile homicide of Marianne Vaatstra spurred new legislation on DNA phenotyping in which race was made into a crucial marker in forensic investigation. In the Vaatstra case, the lack of clues about the identity of the suspect and the availability of good crime‐scene stains had incited the public prosecutor to ask forensic geneticists for help. The prosecutor was particularly interested in the biogeographical ancestry of the unknown suspect. Because the homicide had taken place in the vicinity of a center for asylum seekers, the local population was quick to suspect the inhabitants of the center, leading to a grim and violent situation (Jong and M'charek 2018).

A genetic test was deemed necessary not only to generate a suspect but also to soothe racialized fears and violence against asylum seekers. The forensic geneticist did indeed provide some information: he concluded that the suspect's profile was less common in the Middle East (where many asylum seekers were from) and more prevalent in northwestern Europe and the Netherlands (de Knijff 2006). While the research demonstrated the potential of DNA phenotyping in solving crimes, this technology was at that moment in time, June 2000, forbidden by Dutch law. But the minister of justice responded quickly and introduced a law on externally visible characteristics in early 2003. This law is hence a break with the use of noncoding DNA, also known as “junk DNA,” in forensic DNA profiling.^11^ The promissory nature of DNA phenotyping had produced an exceptional law to regulate technologies that were mostly unavailable (M'charek 2008). It was designed as a so‐called window legislation, a forward‐looking law to regulate DNA phenotyping *avant la lettre*.^12^ This is remarkable, as an often‐heard complaint is that legal progress always lags behind scientific progress. But it was even more remarkable that the law made it literally possible to “determine the *race* of the unknown suspect.”^13^

Although the phenotype or the facial composite that could be sketched in the early 2000s consisted of inferences about sex and race, forensic geneticists were casting a different picture of DNA phenotyping, one that was much more geared toward individuality. A Rotterdam‐based forensic geneticist who received substantial funding to develop this technology for the Dutch forensic practice invited the readers of a popular forensics magazine to imagine the possibility of this technology:

Imagine a world where a near‐perfect likeness could be created from trace DNA evidence collected from a crime scene. This phantom image could be printed, distributed, and used to identify a suspect. (Kayser 2011)

In 2006, the US‐based company DNAPrint Genomics was already promising to deliver the driver's license photo of an unknown suspect. Promoting a product named DNA Witness 2.0, the company stated that this kit would help:

construct a partial physical profile from the DNA and in many cases learn details about the donor's appearance, *essentially permitting a partial reconstruction of their driver's license photo*. (see M'charek 2008)^14^

DNA phenotyping, so the proponents of these technologies argue, is aimed at the identity of the individual. However, in practice it is a clustering technology. It might, for example, tell us that the suspect is a white man with brown eyes and blond hair. Doing so, it produces not an *individual suspect* but a *suspect population*: all white men with brown eyes and blond hair. Nevertheless, given the fact that DNA phenotyping is an emergent technology, and given the promises made about it, one cannot but wonder about the future of this technology. It raises the question of whether the face will eventually be individualized. But a closer look at the investigative practice is productive. The forensic police examiner Charles Jackson (2004) writes:

Does the drawing need to look exactly like the perpetrator to be effective? No, it does not. The likeness should be as accurate as possible, but a general or close likeness will in many cases stimulate recognition on the part of viewers. In contrast to the commonly held belief that highly detailed or photographic images are more effective, these images actually narrow the scope of interpretation on the part of the viewer who simply concludes that they don't know the person in the picture rather than considering the likeness possibilities.

This indicates that the very *incompleteness* of the composite face *is taken as a virtue* in criminal investigation. This is rather counterintuitive, but it makes sense when we understand that these composite faces are meant for public dissemination in the hope that as many people as possible will engage with the images. Their incompleteness captures the gaze and provokes a stream of thought about the possible suspect. Moreover, the fact that this incompleteness produces a suspect population underlines that DNA phenotyping is aimed not at an individual face, such as the face on one person's passport photo, but rather an aggregate face, the composite face of a collective. This face is both general enough to invite responses from a public and specific enough to not include everybody in the population.

To arrive at this specificity, sex and biogeographical ancestry are the most valuable pieces of information. In the practice of a criminal investigation, hints about ancestry will be translated into categories that are relevant in the societies in which a crime is investigated. For example, in the above‐mentioned Vaatstra case, the biogeographical ancestry of the unknown suspect suggested he was of northwestern European or Dutch descent. In the media, this probabilistic statement was translated overnight as “suspect is a white Dutch man.” Similarly, a West African ancestry might have been translated as “suspect is of Cape Verdean descent,” as happened in a Brockton, MA, case in the United States in 2016.^15^

Such practical translations make it possible to focus on concrete groups of people rather than abstract “Western Europeans,” but also help racialize the profile of the unknown suspect. Such translations assume a seemingly unproblematic mapping of biological and sociocultural markers of difference. Furthermore, given the key role played by biogeographical ancestry markers, DNA phenotyping technologies work best when applied to minority populations because they effectively help to reduce the size of the population of interest. For example, the DNA profile in the Vaatstra case that suggested the suspect was probably a white Dutch man did not quite help to narrow down the criminal investigation in the Netherlands. The pragmatics of these technologies and their relevance when they point toward minority populations sets in motion a process of racialization (Jong and M'charek 2018). The profile that is based on *probabilistic* categories has to be translated into *social* categories of people in a particular society with a specific demographic, colonial, or migration history. DNA phenotyping thus becomes entwined with specific sociocultural practices of doing race (Fullwiley 2011).

## TENTACULAR FACES

Giving a face to an unknown suspect can be crucial in crime solving, which obviously aims to find the individual suspect. However, as I have shown, the composite face does not *represent* this individual but rather actively *generates* a suspect. DNA phenotyping technology does not individualize but clusters: it produces a composite face of a collective, one that can direct the police investigator to focus on some groups of people in the population and not others. In addition, precisely because the face is incomplete, it draws the attention of the general public to the crime; it directs their gaze to relate to the face, to specify it.

Given this multiple work of the face, I suggest that the face is better viewed as tentacular, connecting to its surroundings in multiple ways. It reaches out. The notion of *tentacular faces*, I argue, helps us to take seriously this work of connecting and to specify what the face does in practice. In my discussion of Martin's prints, I suggested we view this work as *instructing* the viewer on what to take into account, *fashioning* and *typecasting* what is viewed, and *evoking* feelings and interest in the issue at stake.

I like to think about tentacularity with the help of the work of Deleuze and Guattari. In their essay “Year Zero: Faciality,” they argue that “faces are not basically individual” but semiotic fields that are capable of making socialities (Deleuze and Guattari [1987] 2004, 186). They call this process “facialization,” proposing that: “Concrete faces cannot be assumed to come ready‐made. They are engendered by an abstract machine of faciality (visagéité)” (187). In terms of this faciality machine, there cannot be “any appeal to a preexisting subject” that the face is assumed to represent. The notion of “abstract machine” is used to underline the “social production of face” but also the fact that facialization impacts more than the concrete face. “It performs the facialization of the entire body and all its surroundings and objects” (201). Here, we are reminded of Martin's prints and my suggestion that the face is a material‐semiotic object; this modern abstract machine renders the face into different components that can be measured, compared, taken apart, recombined, quantified, and hierarchized, yet in the end contribute to the face as a whole, a unity. Connecting these parts into wholes makes faces legible, a tool for sorting out differences between categories of people. Deleuze (1986) calls this version of the face the “reflective face.”

But whereas the reflective face expresses common qualities—noses, eyes, and parts that can be compared—the intensive face is about the tendencies and trajectories of the face. The film theorist Rushton (2002, 230) puts it as follows:

The intensive face pulsates, bends, and creeps around its own surface. It is composed of the sum of its parts; that is, instead of the facial unity of the whole being the dominant mode, as it is with the reflective face, in this case the *separate and multiple parts of the face take on a life of their own*. With the intensive face, the whole is subservient to its parts. (emphasis added)

Thus, a single part of the face can facialize the face, the body, the person, and their assumed behavior. “Those eyes,” said Vaatstra's father, looking at a composite drawing of the suspect in the murder of his daughter, “are eyes of a killer. The face is clearly [*duidelijk*] a face of an asylum seeker.”^16^ Focusing on the crucial work done by the parts of the face, Deleuze and Guattari move away from the face as a singular and unified whole. Both the concept of facialization and the concept of intensive face are helpful in elaborating what I mean by the tentacular.^17^

Already as a metaphor, the tentacular disturbs the assumption of a whole, a face that represents, if we think of the tentacular fingers of an organism that might be reaching out in different directions and involved in different work (moving, eating, sensing). It thus helps us to shift the focus to what the face does. This metaphor is furthermore interesting because sensing is precisely its trade. The noun “tentacle” is derived from the Latin *tentaculum*, for feeler, and *tentare*, which means to feel or to try. The tentacular, if we think of the tentacles of a cuttlefish, for instance, is about carefully touching, smelling, grasping, eating, and seeing, but it is also about cautiously withdrawing and pushing away from its environment. Think of a face, moving in and out of the public attention and imagination.

Tentacles are sensory and receptive organs. The grasping and feeding are of particular interest in this context. Forensic composite faces are tentacular because they are active: they move (wander about in public spaces), and they affect and mobilize their public to act. They do so especially because they often index horrible events. Yet, composite faces are also dependent. They are “incomplete” and sketchy, and thus rely on their public to feed them, to fill them in and give them content and contours. Attending to the tentacular means attending to the power of the sensorial (touch, vision, smell, taste). But, crucially, it also means attending to the political power of the affective (Ahmed 2004). Affect, according to Ahmed, does not originate in the individual body; the subject is a “nodal point” (121). Fear can neither be reduced to the object nor the subject of fear but is found in the relation and circulation between them. The following example demonstrates this further and helps me illustrate the relation between face and race in forensic genetics.

**Figure 2:**
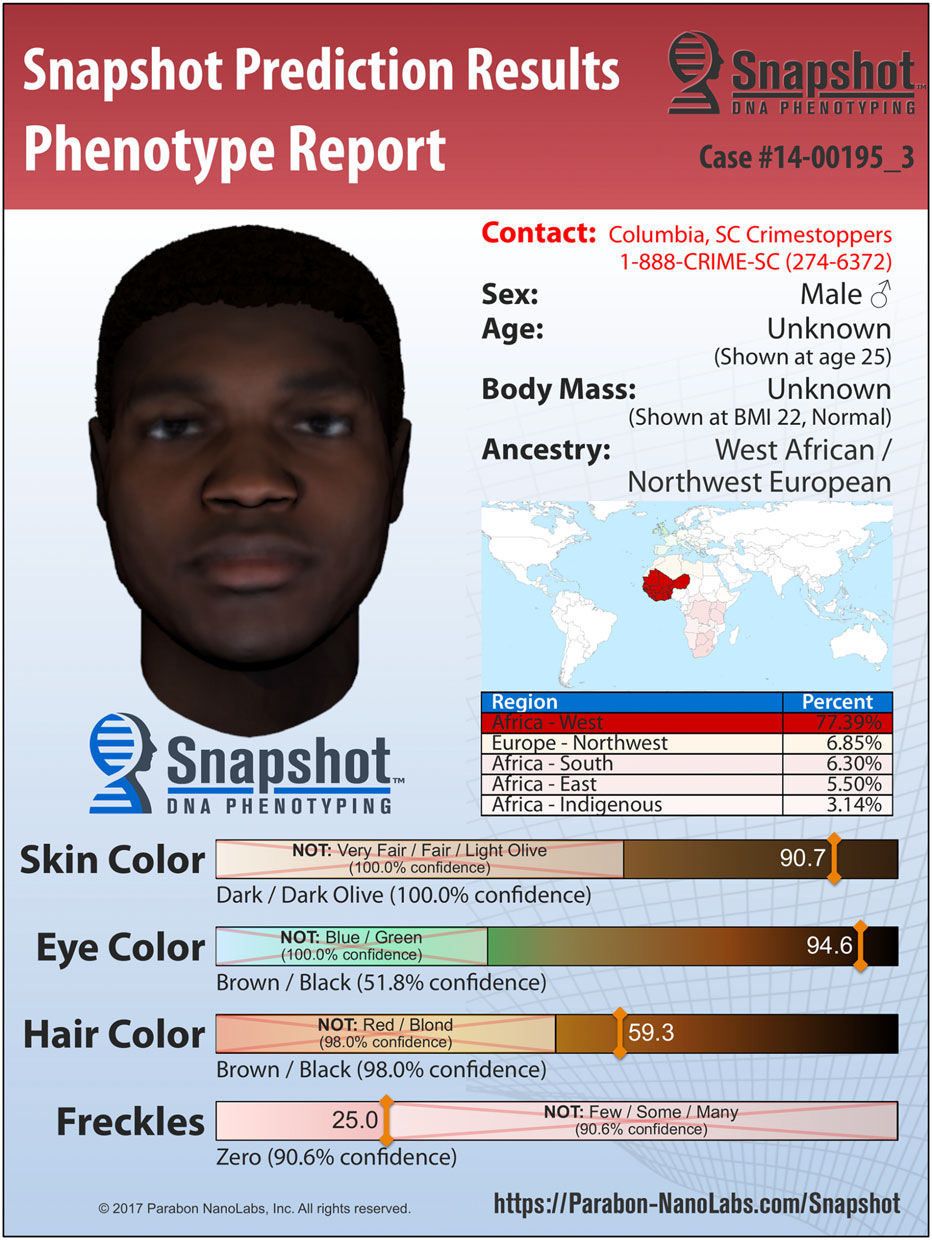
Snapshot of an unknown suspect, released on January 9, 2015, by the police department of Columbia, South Carolina, in the United States. (Courtesy of Parabon. https://snapshot.parabon‐nanolabs.com/posters) [This figure appears in color in the online issue]

Figure 2 is a “Snapshot” of an unknown suspect, released on January 9, 2015, by the police department of Columbia, South Carolina, in the United States. The diagram shows the face of a person whose DNA was found at a crime scene in South Carolina. A mother and her three‐year‐old daughter were gruesomely killed in January 2011. Four years later, there were still no clues about the identity of the suspect. A US company, Parabon, was called to help, and it made its debut with this very Snapshot, presenting its novel DNA phenotyping technology.^18^ The face of the suspect presented here is allegedly based on the DNA found at the crime scene. Mark Vinson, a cold‐case investigator with the police department, said: “We're very hopeful this composite could be the thing that prompts someone to come forward.”^19^ The public was asked to contact Crimestoppers using the number on the diagram. A member of Parabon described their work in producing this face as follows: “Traditional forensic analysis treats DNA as a fingerprint, whereas Snapshot treats it as a blueprint—a genetic description of a person from which physical appearance can be inferred.”^20^ The Snapshot catches our attention. Readable as a portrait, it asks us to consider the suspect's face as singular. However, what if this snapshot is not that different from Martin's prints, discussed above? What if the face does not stand alone but is a material‐semiotic object, enacted through its very relation to all the other attributes surrounding it? From the case number on the top right of the diagram to the various bars indicating traits and probabilities, and the geographical map indicating ancestry, the Snapshot details and disaggregates. So, what precisely are we looking at? What is the face made to be? What does the face do?

The striking thing about the faces produced by Parabon is their portrait‐like character.^21^ The lure of individuality cannot be overlooked. But the DNA evidence contributing to this portrait does not carry further than probabilistic statements about ancestry and skin, eye, and hair color. What does it mean to have 94.6 percent brown or black eyes, with 51.8 percent confidence? How does that translate? The diagram as a whole is relevant and ordered in nontrivial ways. The face of the suspect draws us in, then the graphic elements guide our gaze to the bottom to consider the components of the face—eye, skin, and hair color as well as the possibility of freckles—all detailed in dazzling probabilities found at the level of the molecules, the DNA. But the face does not stop there. It guides us further, from the molecule to the globe, to situate the face and its ancestry (mostly) in West Africa. If one were to doubt this, a table just beneath the map substantiates the claim in more detail, disaggregating and providing statistical backup. Then there is a summary just underneath the contact information of the police, telling us also what we do not know, such as age and BMI, to assure us of the rest of the information. As we study the diagram, we find ourselves moving back and forth from the face to these attributes. It is precisely this movement that validates the face. “Jumping up and down from the gene to the globe” (M'charek 2016), moving from the molecular to the surface solidifies the face of the suspect. We are thus made to engage in the work of *ersehen*; a process is set in motion aimed at learning and recognizing.

But how do we know about the shape of the face or the hairstyle of the suspect? What about the protrusion of the lips and the width of the nose? The “data face” (M'charek 2016) that rolls out of the DNA sequencing machine is given flesh and bones through the geographical location on the map. The ancestry marker, leading to 77.39 percent West African ancestry, is here (together with sex) the key variable. The DNA phenotyping technology works with a database containing 3D pictures of faces and their respective genetic profiles. Based on the ancestry marker of the unknown person, a base face is generated onto which the rest of the genetic markers (skin, eye, and hair color) are added. In this way, ancestry produces a racial type that helps to shape the face of the unknown suspect. Moreover, despite their limited number, the genetic data available help to biologize other elements of the face that cannot be known, such as hairstyle or facial form in rather stereotypical ways. While suggesting individuality, this face is still general and hints in the direction of a group of people instead.

Discussing DNA phenotyping above, I suggested that the incompleteness of the composite face is taken as a virtue. This incompleteness indicates that the aim of this technology is not the face of an individual but that of a collective. Narrowing to a cluster reduces the scope of a criminal investigation from the general population to the “profiled” population. This incompleteness is the basis of the tentacularity of faces. The Snapshot face does not represent the unknown suspect but is active and engaged: it is doing. First, it engages as it instructs the viewer on what to take into account; it educates about the differences that matter. When viewed in isolation, the Snapshot face is fairly general and does not give the viewer much to latch onto. By directing the gaze to the various components and their statistical renderings, it sets a process of contrast and comparison in motion so that its significance can be grasped. Brown or black eyes with 51.8 percent confidence! Second, the face is engaged in fashioning and typecasting what is viewed. While the portrait‐like image is too generic to be informative, it does produce a racial type. But it does not do so in a straightforward manner, because race flickers in and out of existence. Even as the race of the face seems “in your face” because the image represents a person who is supposedly Black, the knowledge presented is probabilistic, and there are no rigid boundaries drawn.

What if the person represented was white? Would we be equally alarmed to examine what happens to race? As viewers, we are activated to engage in the doing of difference. Guiding our gaze from the hair, eye, and skin color to West Africa and back to the image of the face contributes to forging a facial unity. This work of unifying does not crystalize in individuality but is the very fashioning of race, work that happens precisely between what is looked at and the viewer, as well as the context in which this gazing happens.

This context is important: the work of the face evokes feelings and ideas about the issue at stake, in this case a double murder. The case number in the upper‐right corner is a crucial element in this respect. It is evocative of the crime and its specificities as well as of the suspect. “I hope they get him!!!!” was one of the posted responses to the composite face on Facebook.^22^ Evoking emotion, the face also generates engagement with the case. Attention to the case might wither, but the face makes it concrete and keeps the case alive.

So the composite face, a tentacular face, keeps wandering: instructing the viewer, fashioning race, and evoking interest and affect. Intensifying along its routes, it draws a public together on which it can feed in order to be fleshed out. The composite face thus gains content and contours, as to unify and to stop wandering.

## CONCLUSION

In this article, I advocated focusing on what a face can do, and I offered the concept of the *tentacular* to examine that work in practice. This means attending to the politics of the surface. Doing so has allowed me to open up the phenotype for examination. The specific case I focused on is DNA phenotyping, and I have shown how appearance is biologized in the context of forensics via the face, and how this contributes to the doing of race.

The analysis here has focused on the relation between face and race in DNA phenotyping, but one might wonder whether it would be possible to do the face differently in that practice and beyond—or, alternatively, whether it would be possible to do the face in ways that do not necessarily enact race. One of the problems is that current practice of DNA phenotyping relies heavily on biogeographical ancestry for making sense of its data. This is in fact a broader problem in the context of population genetics and big data, where biogeographic ancestry has become a category of value, as it helps to tease out information from vast amounts of data (see also Duster 2015; Kahn 2013). This use of “race” as a tool, a heuristic of sorts, contributes to the reification of race as an object out there in the world. It does so even more forcefully as this information travels from science to society, where it hooks into other received racialized categories and so strengthens the assumed validity of race as a biological category. This trend is chilling and disappointing, as current population genetics has the potential to undo racialized categories and contribute to a much more nuanced and intricate account of human history and relations (Serre and Pääbo 2004).

An important scholarship on race in the life sciences has focused on the molecularization of difference and on the genotype. I call here for more attention on the phenotype and the biologization of appearance, not only because it is gaining relevance in the life sciences but also because we lack a vocabulary to talk about the phenotype in relation to race. Now, because talking about the body, biology, and race risks reifying differences and essentializing race, we must attend to the phenotype as a relational phenomenon—a phenomenon that is not locked up in the body but comes about in practices, as a relation between bodily markers or facial characteristics and other elements that surround the body, and a specific sociopolitical context in which these relations come about. But my claim about the tentacularity of face is still broader. I want to suggest that all faces are active, demanding, and tentacular. When we encounter another person on the street, that person's face will set a process in motion connecting parts of the face to clothing, the rest of the body, other bodies, buildings, and so on, as to make the face readable. While this work is part of life, it is not value‐free or apolitical. The work of the face is the production of appearance, and this may turn into “phenotypic othering” (M'charek et al. 2014), the racialization of specific groups of people based on a heightened visibility in specific political situations.

This is not to say that there are no differences between actual faces and representations thereof. For example, the face encountered on the street is usually accompanied by much more information, and it is changing in time. Yet, approached through the methodological lens of tentacularity, actual and represented faces become comparable, as we can study what these faces do and how they implicate the observer in doing so. Not in general, but in specific practices, both can be examined to learn about how they instruct the viewer by reading (parts of) the face in relation to its surrounding, fashion and typecast differences by contrasting this face to others as to cluster it, and evoke feelings and interest, prompting specific modes of relating. The face and its tentacularity thus deserve more attention as we strive to understand the politics of race in practice.

## Footnotes

1 Given my interest in the relation between face and race, I am frequently referred to the work of Emmanuel Levinas by colleagues. Although I find his work important for thinking about ethics and the politics of othering, it is less suited for opening up the face for anthropological, empirical analysis.

2 This article is part of larger research project, the RaceFaceID project (http://race‐face‐id.eu), in which we study three forensic technologies of face‐making: (1) genetic facial phenotyping and the inference of visible traits from DNA; (2) craniofacial reconstruction; and (3) facial composites based on eyewitness accounts. We study these technologies of face‐making in order to understand how race figures and comes about in specific practices. Following the relation between the individual and the population across different forensic sites (laboratories, police stations, the courts, and the media), we are interested in finding out when and how this relation becomes racialized.

3 Exactly how and where these multiple histories resonate and inform contemporary practices is the focus of the RaceFaceID research project.

4 We know from the work of early anthropology, ethnology, and ethnography that, in search of racial types, biological and cultural markers were both collected: biology alongside differences in food, environment, language, religion, etc. This mixture of nature and culture is at the heart of Martin's physical anthropology as well (see Keller 2006).

5 See: http://www.the‐scientist.com/?articles.view/articleNo/12937/title/The‐Human‐Genome/.

6 See: https://allofus.nih.gov.

7 I obviously play with face and surface, but the much more substantial point concerns the history of physical anthropology, where the study of human variation was much more about the surface of the body and its environment.

8 Although skin color is problematized in critical race studies, the focus there is on the meanings (legal or social) attached to color.

9 In this article, I do not elaborate on the crucial relation between race and sex—that is, how race gets done through a focus on sex differences and the other way around. Doing so goes beyond the scope of my argument here, but on the coproduction of sex and race, see the classical example of Sara Baartman and her ante‐ and postmortem examination by George Cuvier and Henri de Blainville (Fausto‐Sterling 1995).

10 The sex of the unknown suspect is routinely determined by a so‐called Amelogenin test, a test that differentiates between the sets of sex chromosomes: XX for female and XY for male. It does not come as a surprise that this genetic distinction does not necessary meet the sexual identity of unknown persons—not only due to medical conditions, such as the Swyer Syndrome or the Turner Syndrome, which results in women carrying an XY chromosome pair, but also because some person might prefer to pass as a gender that is not typically associated with their genetic sex, such as transgender or in drag. In 2004, in a homicide case in the Netherlands, the police were looking for the unknown suspect. Based on Y‐chromosomal research, the suspect was a man of Western descent. In 2005, a woman who was born as a boy became the main suspect. Although she was interrogated in 2004, she was not considered a suspect, as her gender did not match the DNA profile (see Toom 2011).

11 In the controversial early days of forensic research, the fact that DNA profiles were based on noncoding DNA was presented as less invasive to the individual, as this part of the DNA does not disclose any information about genetic predisposition and so protects people's right not to know about potential genetic diseases that they might carry.

12 In a sense, this legislation provides an interesting example of Fortun's (2005) take on promissory science, a science that is productive in a bewildering and surprising way and that should not be reduced to specific promises.

13 See Besluit DNA‐onderzoeken in Verband met het Vaststellen van Uiterlijk Waarneembare Persoonskenmerken.Staatsblad. 2003; 201: pp. 1–2. See also M'charek (2008).

14 See Ted Kessis at http://www.bioforensics.com/conference04.Racial_dentification/index.html.

15 See https://www.enterprisenews.com/news/20170307/will‐sketch‐help‐catch‐this‐brockton‐rapist. See also Sauer (1993, 82) on the convenience of such translations in the communications between physical anthropologists and law enforcement.

16 Vaatstra's father took part in a television show called the 6th Senses wherein psychics were asked to help the forensic artist. See https://www.youtube.com/watch?v=cf6Oi_W7t5I. Despite the fact that Y‐chromosomal analysis suggested that the subject's descent was probably Dutch, the public, including Vaatstra's father, kept suspecting the residents of the asylum‐seeker center.

17 See also Haraway (2016) on the notion of “tentacular thinking” and the concept of the Chthulucene as a way out of environmental catastrophes, by gathering stories that help us to think surprising connections across species.

18 The DNA phenotyping technology of Parabon relies on scientific work done by Peter Claes, Mark Shriver, and colleagues (2014). Although this technology is presented as one that leaves the racial history of facial landscaping behind, in practice it relies heavily on classical racial anthropology and the racial classification of faces into well‐known categories.

19 See https://www.fox5dc.com/news/new‐dna‐technique‐may‐reveal‐face‐of‐killer‐in‐unsolved‐double‐murder.

20 See https://www.fox5dc.com/news/new‐dna‐technique‐may‐reveal‐face‐of‐killer‐in‐unsolved‐double‐murder.

21 This incidentally is one of the reasons Parabon is criticized: while the faces they produce are more or less generic faces, they also draw on the genre of the portrait, and in doing so, evoke individuality. DNA phenotyping is often likened, in this regard, to forensic sketching. However, forensic artists, including sketch artists and cranio‐facial reconstructionists, make important distinctions between faces that are meant for investigatory purposes and those meant for public circulation in museums or otherwise. These artists are typically weary of aesthetics and details unsupported by evidence, as both would detract from the accuracy and usability of their production.

22 See https://www.facebook.com/search/top/?q=double%20murder%20carolina%20parabon%20snapshot.
